# Associations between motor competence and physical activity levels of children with intellectual disabilities and/or autism spectrum disorder: Movement matters

**DOI:** 10.1177/17446295231203764

**Published:** 2023-09-20

**Authors:** Sarah L Taylor, Samantha J Downs, James R Rudd, Bronagh McGrane, Craig A Melville, Arlene M McGarty, Lynne M Boddy, Lawrence Foweather

**Affiliations:** Physical Activity Exchange, Research Institute of Sports and Exercise Sciences, 4589 Liverpool John Moores University, Liverpool, UK; 25567 Norwegian School of Sport Sciences, Oslo, Norway; 8817 Dundalk Institute of Technology, Dundalk, Ireland; School of Health & Wellbeing, 3526 University of Glasgow, Glasgow, UK; Physical Activity Exchange, Research Institute of Sports and Exercise Sciences, 4589 Liverpool John Moores University, Liverpool, UK

**Keywords:** autism spectrum disorder, children, intellectual disability, motor competence, physical activity

## Abstract

Motor competence is important for lifelong physical activity (PA). The current study aimed to examine associations between PA and motor competence. In total, 43 children aged 7–12 years with intellectual disabilities and/or autism spectrum disorder completed anthropometric measures, the Bruininks-Oseretsky Test of Motor Proficiency-2, and wore a wrist accelerometer to capture total PA, moderate-to-vigorous PA (MVPA), average acceleration, and intensity gradient. No significant associations were found between PA outcomes and motor competence. Motor competence performance was commonly ‘below average’ or ‘average’. The weakest subtests were upper limb coordination and strength. The strongest subtest was running speed and agility. Total weekly MVPA was 336.1 ± 150.3 min, higher than UK recommendations of 120-180 per week for disabled children and young people. Larger scale studies are needed to better understand the relationship between PA and motor competence. Future research should also consider the influence of environmental factors on PA in this group.

## Background

The term intellectual disabilities refers to, and encompasses a range of conditions including, global developmental delay and Down syndrome, characterised by significant limitations with intellectual functioning and adaptive behaviour (Schalock et al., 2021). Autistic Spectrum Disorder (ASD) covers a group of neurodevelopmental disorders characterized by presence of social, communication and interaction difficulties as well as restricted repetitive behaviours (Faras et al., 2010). Deficits observed in ASD can present with more sever forms of intellectual disabilities such as taking longer to understand information (Thurm et al., 2019). Furthermore, individuals with both ASD and intellectual disabilities appear to have a common genetic aetiology, with up to 50% of the children with ASD thought to have comorbid intellectual disabilities (Totsika et al., 2011).

Participation in physical activity (PA) contributes to the improvement, or at least the maintenance, of physical and psychosocial health amongst children with intellectual disabilities and ASD (Kapsal et al., 2019; Smith et al., 2022). PA guidelines from the World Health Organization (WHO, 2020) state that children with disabilities should achieve an average of 60 minutes of moderate to vigorous PA (MVPA) per day across the week, “where possible”, to accrue these benefits, which is consistent with the WHO PA guidelines for typically developing (TD) children. In the UK, specific PA guidelines for disabled children and young people have recently been published. These differ in recommending 20 minutes of PA per day or a total of 120 to 180 minutes of aerobic PA per week at a moderate-to-vigorous intensity (Department of Health and Social Care, 2022; Smith et al., 2022).

Many children with intellectual disabilities and/or ASD are insufficiently active and face numerous barriers to PA participation (Case et al., 2020; McGarty and Melville, 2018). Health markers such as low fitness levels and high rates of overweight and obesity evident in this population could be improved through increased PA participation (Maïano et al., 2016; McCoy et al., 2016; McCoy and Morgan, 2020; O’ Shea et al., 2018; Wouters et al., 2020). Thus, understanding factors that may influence PA behaviours and developing ways to address and overcome known barriers among these populations is of great importance for long-term health promotion.

Motor skill competence is the mastery of common fundamental movement skills and goal-orientated movements that include large muscle groups or the whole body (Gao et al., 2021; Robinson et al., 2015). Motor competence is an essential foundation on which to build more complex movement skills, and therefore is not only an influential factor, but a necessity for lifelong PA participation (Hulteen et al., 2018; Logan et al., 2018). The relationship between PA and motor competence is both reciprocal and longitudinal across childhood and adolescence in that physically active children have more opportunities to improve motor competence while improved motor competence can lead to increased success and enjoyment and therefore increased PA engagement (Barnett et al., 2022; Lima et al., 2017). Thus, the association between motor competence and PA is considered dynamic and hypothesized to strengthen over time (Stodden et al., 2008). Further, a positive relationship between motor competence and cardiorespiratory fitness, as well as an inverse relationship with weight status has been evidenced (Lubans et al., 2010).

Children with intellectual disabilities and/or ASD demonstrate lower levels of motor competence than would be expected both for their age norms and in comparison, to their TD peers (Gkotzia et al., 2017; Pitetti et al., 2017). Significant differences between TD children and children with intellectual disabilities have been found across locomotion, object manipulation and balance skill categories (Kavanagh et al., 2023). These impairments in movement skill ability when compared to TD peers are also evident in children with ASD when IQ scores are controlled for, meaning that cognitive abilities alone cannot account for the differences (Gandotra et al., 2020). Children with ASD have been shown to have fine motor skill deficits (Matson et al., 2011; Mohd Nordin et al., 2021). Although fine motor skills do not fall under the umbrella of foundational movement skills (e.g., running, throwing, kicking) and thus are not considered to play a significant role in the initiation, maintenance or decline of PA (Hulteen et al., 2018; Stodden et al., 2008). As evidence suggests that children with intellectual disabilities and/or ASD are less active than their TD peers and have lower motor competence, studies to examine the relationship between motor competence and PA in this population are warranted.

While several research studies have documented positive associations between motor competence and PA participation in TD children and adolescents (Duncan et al., 2022; Holfelder and Schott, 2014; Lopes et al., 2019), relatively little is known about the relationship between motor competence and PA in children with intellectual disabilities and/or ASD. This relationship may be more complex because of impairments associated with intellectual disabilities and/or ASD, such as difficulties in processing and responding to information, which can cause issues with poor cognitive proficiency and motor development delay (Jeoung, 2018; Juntorn et al., 2017; Mohd Nordin et al., 2021). A recent systematic review of the correlates of PA in children and adolescents with intellectual disabilities found that having better motor development was positively associated with PA (Sutherland et al., 2021). However, this positive association was demonstrated by only two studies, indicating a lack of evidence (Eguia et al., 2015; Wouters et al., 2019). The study by Eguia et al. (2015) was conducted in the Philippines and examined whether fundamental movement skill components (locomotor, object control) were associated with PA levels (pedometer assessed daily step count) in 60 children with intellectual disabilities aged 5–14 years. Significant positive associations were found between movement skills and PA, with object control skills (catch, overhand throw, underhand roll, stationary bat, stationary dribble, kick) accounting for 26.7% of overall daily step count variance (Eguia et al., 2015). Chu and colleagues found waist-worn accelerometer assessed MVPA to be positively related to coordination, strength, and agility in a Chinese sample of 63 male adolescents with ASD aged 12-18 years (Chu et al., 2020). In another Chinese sample, Wang et al. (2022) examined 93 children and adolescents with intellectual disabilities aged 8-17 years. Object control skills were found to be a significant predictor of waist-worn accelerometer assessed MVPA for boys and locomotor skills to be a significant predictor of MVPA for girls. Overall, the limited evidence available indicates that movement competence is positively associated with PA in children with intellectual disabilities, though more research is required.

Absent or unclear methodological reporting of PA and motor competence assessments and procedures limit our ability to draw comparisons between studies and precludes interpretations of the available evidence. Generally, the selection of the motor competence assessments used in studies are not based on population specific evidence for validity, reliability, and feasibility (Downs et al., 2020; McGarty et al., 2018). Similar issues arise when considering the measurement of PA in the target population. A review by Leung and colleagues (2017) demonstrated that there is a lack of standardised protocols when using accelerometers to measure PA among children and adults with intellectual disabilities. For example, the lack of consistency in accelerometer cut points used to determine intensity thresholds for PA makes it difficult to compare PA levels of children with intellectual disabilities and/or autism across different studies. This has resulted in suggestions that raw accelerations are used, transitioning away from proprietary accelerometer metrics (i.e., counts) to allow for comparable metrics between studies (Fairclough et al., 2023). Examples of these non-proprietary outcomes includes accelerations due to movement representing activity volume and a profile of PA intensity, which have been shown to be independently related to a range of health and wellbeing outcomes (Fairclough et al., 2023; Rowlands, 2018). These recently introduced accelerometer metrics are yet to be studied in a population of disabled children.

The motor competence of children with intellectual disabilities and/or ASD is not fully understood and consequently the associations between PA and motor competence in these populations are also unknown. Therefore, the aim of this study was to assess PA (including volume and intensity) and motor competence levels of children with intellectual disabilities and/or ASD and to examine the associations between PA and motor competence. It is hypothesised that motor competence levels will be below average in comparison to TD normative data, and that better motor competence will be associated with higher PA.

## Methods

Data for the current analyses has been combined from two separate studies, using the same methods outlined below. Data were collected in study one between November 2019 and January 2020. Data were collected in study two between March 2022 and July 2022. Twenty-one special educational needs (SEN) schools (9 primary, 6 secondary, 6 primary/secondary) in the northwest of England were invited to take part in the study. SEN schools were invited via gatekeepers at the local authority PA and sports development department and the regional active partnership organisation, who circulated study information packs to relevant schools in the northwest region. Gatekeeper consent was obtained from six schools across the two studies. Two schools (primary level) provided gatekeeper consent for and participated in both study one and study two with different cohorts of children participating in each study meaning data was not included from the same children twice (29% recruitment rate; n = 2 primary; n = 1 secondary; n = 1 with primary and secondary aged pupils). Reasons for not participating in the study were not provided by the schools.

Ethical approval for each study was obtained from the Liverpool John Moores University Research Ethics Committee (ref no’s: 19/SPS/007; 21/SPS/040). Collectively, n = 290 pupils were invited to take part in these studies via information packs distributed by class teachers. Written consent was required from parent/carer(s) and written assent was required from children. Additional verbal assent was also requested from children before they participated in any measures. Participant demographic information including, home postcode, child’s ethnicity, date of birth, medical related family history, general health data, and disability diagnosis was collected from the parent/carer consent forms and questionnaire. Home postcodes were used to establish neighbourhood-level socioeconomic status. Indices of Multiple Deprivation (IMD), a UK Government produced deprivation measure for England, provided rank scores, where decile one represents the most deprived 10% of areas nationally (The English Indices of Deprivation 2015).

Stature was assessed to the nearest 0.1 cm using a portable stadiometer (Leicester Height Measure, Seca, Birmingham, UK). Body mass was assessed to the nearest 0.1 kg (761 scales, Seca). Body mass index (BMI) was calculated as body weight in kilograms divided by height in metres squared for each participant. BMI z-scores were assigned (Cole et al., 1995) and age and sex specific BMI cut points established participants as underweight, normal weight or overweight/obese (Cole et al., 2000).

During the regular school term in each study, and thus representative of usual free-living activity, all participating children wore an ActiGraph GT9X triaxial accelerometer (ActiGraph, Pensacola, FL, USA) on their non-dominant wrist for seven consecutive days. Further instructions included wearing the accelerometer at all times (24 h·day^1^), except when engaging in water-based activities such as bathing and swimming. This 24h protocol was a recommendation rather than requirement, due to potential sensory issues with some children who would subsequently remove the accelerometer more often, including during sleep. The device was initialised to record raw accelerations at a frequency of 100 Hz. Using ActiLife version 6.13.4 (ActiGraph), data were downloaded from the devices, saved as GT3X files and then converted to CSV format. Raw data files were processed in R (http://cran.r-project.org) using GGIR. This processing within GGIR converted raw tri-axial accelerometer signals into one omnidirectional measure of acceleration, referred to as the Euclidean norm minus one (ENMO; van Hees et al., 2013, 2014). ENMO values were averaged per 1 s epoch over each of the seven monitored days (Fairclough et al., 2016, 2022). This average ENMO value represents acceleration due to movement, corrected for gravity and is measure of activity volume, expressed in mg (Rowlands, 2018). Additionally, detection of implausible values and detection of non-wear time was imputed by default in GGIR (van Hees et al., 2013). The only available published ENMO prediction equations (from a non-disabled sample) were used to identify cut-points for classifying sedentary time (50 mg; Hurter et al., 2018) and activity as MVPA (3 metabolic equivalents (METs; child-specific); 200 mg; (Hildebrand et al., 2014). Minimum wear time to be included in the analysis was set to 9 h for a minimum of two weekdays and one weekend day (Fairclough et al., 2016; Foweather et al., 2015). A standardized waking hours day of 7 am to 11 pm was used to account for participants who may not have followed the 24 h protocol (Fairclough et al., 2022). In addition to average acceleration and time spent in light, moderate and vigorous PA, the profile of PA intensity, termed the Intensity Gradient (IG; Rowlands, 2018) was explored for the established waking hours. This outcome was calculated following the method described by Rowlands et al. (2018). The IG value is always negative as it is reflective of the drop in time accumulated in increasing intensity, thus a steeper drop and more negative value represents a lower intensity profile compared to a shallower drop and less negative value (Rowlands et al., 2018).

The Bruininks-Oseretsky Test of Motor Proficiency-2 (BOT-2) testing battery was used to assess motor competence and administrated by trained researchers (Bruininks and Bruininks, 2005). A systematic review of motor competence assessments for use amongst children with intellectual disabilities and/or ASD has identified the BOT-2 tool as the most valid, reliable, responsive and feasible for use within this population (Downs et al., 2020). For this study, six out of eight subtests were used. The subtests selected were: manual dexterity, upper-limb coordination, bilateral coordination, balance, running speed agility, and strength. These subtests make up three motor composites; manual coordination, body coordination, and strength and agility. The two subtests not used were the fine motor precision and fine motor integration which form the fine manual control composite. As the focus of the study was the relationship of gross motor skills with PA, measurement of these fine motor skills was out of scope for the study aims.

To avoid any lapses in concentration and challenging behavior, in many cases the test was administered across 2-4 sessions. Therefore, administration occurred across 15-30 minutes segments with up to three separate days of testing required. A trained member of the research team physically demonstrated each task to every child before completion and the BOT-2 administration easel containing large colour photos of a child performing the items was visible for each child to view (Deitz et al., 2007). After completing the necessary assessments, each participating child was scored in accordance with the BOT-2 manual (Bruininks and Bruininks, 2005). Each child received a raw score for each assessment, which was then converted to a point score. Using the BOT-2 reference values, sex specific age equivalents for point scores were obtained, providing the indication of descriptive categories (average, below average, above average).

Data were analysed using IBM SPSS Statistics Version 28 (IBM Corporation, New York). Statistical significance was set at p < 0.05. Firstly, data were explored and checked for normality. Descriptive statistics were calculated for participants, grouped by sex and disability (intellectual disability only, ASD only or dual diagnosis of intellectual disability and ASD) and were reported as means (±SD). Sex differences in age, BMI, BMI z-scores, whole week, weekday and weekend day average PA variables and motor competence summary variables were examined using independent t-tests. Differences in the same outcomes between disability groups were examined using one way ANOVA. Multilevel mixed linear regression models were used to examine associations between motor competence and PA, with average total PA, MVPA, ENMO and intensity gradient entered as the outcome variables and motor competence (1. Total skill score. 2. Motor competence skill composites; manual coordination, body coordination and strength & agility. 3. Motor competence skill subtests; manual dexterity, upper-limb coordination, bilateral coordination, balance, running speed & agility and strength) as the predictor variables, resulting in three models for each outcome variable (12 in total). All models were adjusted for age, sex, BMI-z, and accelerometer wear time. Disability type and disability severity were added to the models but did not significantly improve the fit and thus were not included as covariates. Adjustment for school level clustering also did not significantly improve the fit and thus was not undertaken.

## Results

Returned signed parent/carer consent and child demographic and assent forms were received for 67 pupils with intellectual disabilities and/or ASD (23% participation rate), of which 43 children (64% of the recruited sample; 39 boys; 91%) aged 7-12 years (M 9.5, SD 1.2) completed all assessments and were included in the analysis. Descriptive statistics are presented in Table 1. Out of the three disability groups (intellectual disabilities only, ASD only, combined intellectual disabilities and ASD) the ASD only group had the highest number of children within the sample (n=21). A large proportion of children were from the most deprived areas of England, with 42% (n=18) of children ranked on the highest decile of deprivation compared to 5% (n=2) of children who were ranked on the lowest decile of deprivation. Most of the children were White British (98%, n=42), with one participant (2%) of White and Black African descent. Over half of the children were normal weight (58.1%), with over a quarter either overweight (16.3%) or obese (9.3%) and the remaining 16.3% classed as underweight.

**Table 1.** Descriptive statistics for age, anthropometry, physical activity and motor competence skills.

	Group (*n* = 43)		Boys (*n* = 39)		Girls (*n* = 4)		Intellectual Disability (*n* = 8)		ASD (*n* = 21)		Combined (Intellectual Disability & ASD) (*n* = 14)	
	Mean	SD	Mean	SD	Mean	SD	Mean	SD	Mean	SD	Mean	SD
Age (yrs)	9.5	1.2	9.4	1.2	10.1	1.4	9.6	1.3	9.4	0.8	9.6	1.6
BMI (kg/m^2^)	17.8	3.5	17.5	3.1	20.8	6.1	16.7	3.9	18.0	3.7	17.8	3.5
BMI-z	0.2	1.4	0.2	1.5	0.3	1.1	0.1	1.4	0.2	1.3	0.3	1.7
Accelerometer output variables												
Wear time (min/day)	1234.3	204.9	1239.4	204.4	1184.1	233.9	1299.3	163.6	1189.3	215.2	1264.6	207.6
Whole Week Total PA (min/day)	218.8	44.7	220.6	45.7	200.6	32.6	229.2	37.0	221.6	44.7	208.5	49.5
Whole Week MVPA (min/day)	56.7	20.4	58.4	20.3	40.1	15.6	61.2	19.3	55.5	17.7	55.8	25.5
Weekday Total PA (min/day)	224.7	48.0	226.7	47.0	205.3	61.3	233.1	33.5	228.8	47.1	213.7	56.8
Weekday MVPA (min/day)	59.5	22.2	61.1	22.0	44.5	21.3	63.6	20.3	59.0	20.0	58.0	27.3
Weekend Total PA (min/day)	205.0	64.4	207.3	67.2	181.9	10.6	220.8	54.4	208.2	64.3	191.0	71.2
Weekend MVPA (min/day)	50.1	22.6	52.1	22.7	30.4	8.5	56.1	18.6	48.3	19.2	49.3	29.4
Average acceleration (m*g*)	60.7	18.6	61.9	18.8	49.2	13.3	62.2	16.9	59.8	14.8	61.3	25.0
Intensity Regression line												
Intensity Gradient	−2.07	0.16	−2.06	0.15	−2.17	0.25	−2.10	0.16	−2.07	0.15	−2.05	0.19
Constant	12.7	0.70	12.7	0.69	12.9	0.94	12.9	0.71	12.8	0.77	12.5	0.57
Explained variance (*R*^2^, %)	94.4	0.04	94.4	0.04	94.3	0.11	94.2	0.01	95.1	0.05	93.3	0.02
Motor Competence variables												
Total skill score (0–239)	151.4	30.3	150.6	31.5	158.8	16.7	155.8	35.6	145.9	33.9	157.1	20.6
Motor competence skill composites												
Manual Coordination composite (0–84)	50.6	12.7	50.4	13.1	53.0	8.0	53.5	14.5	49.5	13.2	50.6	11.4
Body Coordination composite (0–61)	51.2	7.6	50.9	7.8	53.8	4.7	51.3	6.1	49.4	9.2	53.8	4.7
Strength and Agility composite (0–94)	49.6	13.1	49.3	13.7	52.0	7.1	51.0	16.5	47.0	14.5	52.7	8.1
Motor competence skill subtests												
Manual Dexterity (0–45)	10.9	3.7	11.2	3.6	8.5	3.8	11.2	5.0	10.9	4.0	10.7	2.6
Upper-Limb Coordination (0–39)	10.3	3.6	10.2	3.4	10.5	5.4	11.4	3.1	10.3	3.8	9.7	3.6
Bilateral Coordination (0–24)	14.3	4.6	14.4	4.6	14.0	4.5	15.6	3.6	13.8	4.5	14.7	5.4
Balance (0–37)	13.5	3.9	13.7	4.0	12.0	3.8	11.4	3.4	13.5	3.9	14.5	4.2
Running Speed & Agility (0–52)	15.5	4.7	15.6	5.0	14.8	1.5	14.6	3.2	13.5	3.9	14.5	4.2
Strength (0–42)	10.6	5.0	10.5	4.8	11.0	6.8	13.8	8.7	9.7	4.2	10.4	3.9

Abbreviations: BMI, body mass index; PA, physical activity assessed by accelerometry; MVPA, moderate-to-vigorous PA; Wear time, accelerometer wear time; ASD, autistic spectrum disorder.

Total MVPA across the week was 336.1 ± 150.3 minutes, considerably higher than the UK recommendations of 120-180 per week for disabled children and young people, of which 41 out of 43 participants achieved (95.3%). Conversely, less than half (42%) the children in current study met the more widely recognised PA guidelines of 60 minutes per day which are not specific for disabled children and young people. Children engaged in significantly greater amounts of PA on weekdays (total PA 224.7 ± 48.0 minutes/day, MVPA 59.5 ± 22.2 minutes/day) compared to weekend days (total PA = 205.0 ± 64.4 minutes/day, MVPA 50.1 ± 22.6 minutes/day, MVPA difference, p = 0.002). When comparing between sex and disability groups, there were no significant differences in PA or motor competence levels.

Motor competence performance of the sample was most commonly categorised as ‘below average’ or ‘average’ based on TD normative data included in the BOT-2 manual. The weakest subtests were upper limb coordination (n=23 / 53% ‘below average’; n=20 / 47% ‘average’) and strength (n=23 / 54% ‘below average’; n=18 / 42% ‘average’). The strongest subtest was running speed and agility (n=9 / 21% ‘below average’; n=28 / 65% ‘average’; n=6 / 15% ‘above average’). Results for the remaining subtest were as follows: manual dexterity (n=20 / 47% ‘below average’; n=22 / 51% ‘average’; and n=1 / 2% ‘above average’); bilateral coordination (n=10 / 23% ‘below average’; n=24 / 56% ‘average’; n=9 / 21% ‘above average’) and balance (n=11 / 26% ‘below average’; n=28 / 65% average; n=4 / 9% ‘above average’).

Table 2 shows the associations between the motor competence predictor variables and the outcomes of total PA and MVPA. Total skill score was not associated with total PA (p = 0.8) or total MVPA (p = 0.53). No associations were observed between any of the motor competence composites or subtests and the total PA and MVPA outcomes. Table 3 shows the associations between the motor competence predictor variables and the outcomes of average ENMO and IG. Total skill score was not associated with average ENMO (p = 0.46) or intensity gradient (p = 0.31). No associations were observed between any of the motor competence composites or subtests and the ENMO and IG outcomes.

**Table 2.** Summary of mixed regression analyses for total PA and MVPA outcomes.

	Total PA					MVPA				
	B	SE B	LCI	UCI	*p* value	B	SE B	LCI	UCI	*p* value
Total Skill Score	−0.07	0.20	−0.48	0.34	.74	0.06	0.09	−0.13	0.25	.55

Manual Coordination	0.91	0.89	−0.88	2.7	.31	0.39	0.42	−0.45	1.22	.36
Body Coordination	0.72	1.15	−1.60	3.03	.53	0.44	0.54	−0.64	1.52	.42
Strength & Agility	−1.32	0.97	−3.28	0.63	.18	−0.43	0.45	−1.34	0.49	.35

Manual Dexterity	1.06	1.81	−2.60	4.72	.56	0.08	0.85	−1.64	1.80	.93
Upper-Limb Coordination	0.92	1.35	−1.80	3.63	.50	0.61	0.63	−0.67	1.88	.35
Bilateral Coordination	1.91	1.65	−1.41	5.23	.25	0.22	0.77	−1.34	1.78	.78
Balance	−0.22	1.96	−4.17	3.73	.91	0.99	0.92	−0.87	2.85	.29
Running Speed and Agility	−2.50	1.31	−5.14	0.14	.06	−0.73	0.62	−1.97	0.51	.24
Strength	−0.01	1.37	−2.77	2.76	1.00	−0.23	0.64	−1.53	1.07	.73

Note: *B*, beta; SE *B*, standard error beta; 95% CI, confidence interval; L, lower; U, upper; Total PA, Total physical activity time; MVPA, time spent in moderate-to-vigorous intensity PA; All models were adjusted for age, sex, BMI z-score, accelerometer wear time.

**Table 3.** Summary of mixed regression analyses for average ENMO and intensity gradient outcomes.

	Average ENMO					Intensity Gradient				
	B	SE B	LCI	UCI	*p* value	B	SE B	LCI	UCI	*p* value
Total Skill Score	0.07	0.09	−0.11	.024	.46	0.00	0.00	−0.00	0.00	.29

Manual Coordination	0.41	0.39	−0.36	1.19	.29	0.01	0.00	−0.00	0.01	.12
Body Coordination	0.31	0.50	−0.69	1.31	.53	−0.00	0.01	−0.10	0.01	.87
Strength & Agility	−0.36	0.42	−1.21	0.49	.39	−0.00	0.00	−0.10	0.01	.53

Manual Dexterity	0.13	0.79	−1.47	1.73	.93	−0.00	0.01	−0.2	0.01	.77
Upper-Limb Coordination	0.60	0.59	−0.58	1.79	.31	0.01	0.01	0.00	0.02	.054
Bilateral Coordination	−0.12	0.72	−1.56	1.33	.87	−0.01	0.01	−0.02	0.00	.20
Balance	1.05	0.85	−0.67	2.77	.23	0.01	0.01	−0.01	0.02	.23
Running Speed and Agility	−0.49	0.57	−1.64	0.66	.39	0.00	0.01	−0.01	0.01	.98
Strength	−0.35	0.60	−1.56	0.85	.56	−0.01	0.01	−0.02	0.00	.17

Note: *B*, beta; SE *B*, standard error beta; 95% CI, confidence interval; L, lower; U, upper; Average ENMO, average acceleration (a measure of activity volume); Intensity Gradient, the profile of PA intensity; All models were adjusted for age, sex, BMI z-score, accelerometer wear time.

## Discussion

This novel study examined the associations between PA and motor competence of children with intellectual disabilities and/or ASD and explored overall PA and motor competence levels. Furthermore, this is the first study to investigate and replicate the use of average acceleration and IG in this population. Results showed that, controlling for age, sex, BMI-z, and accelerometer wear time, there were no significant relationships between PA and motor competence; this was found when exploring total PA, MVPA, average acceleration, and IG with total motor competence, motor competence composites and motor competence subtests. Motor competence performance was most commonly ‘below average’ or ‘average’. Total MVPA across the week was 336.1 ± 150.3 minutes. The amount of MVPA accrued on weekdays (59.5 ± 22.2 minutes) was significantly higher than on weekend days (50.1 ± 22.6 minutes; p = 0.002).

No associations were found between motor competence and accelerometer measured PA in the present study. There is very limited previous research to compare our findings with. The only comparable study published included a sample of n = 93 children and young people with intellectual disabilities aged 8-17 years from western China and reported contrasting findings (Wang et al., 2022). Results showed a significant positive correlation between MVPA and total fundamental movement skill proficiency assessed using the Test of Gross Motor Development 2 (TGMD-2), with an r value of 0.556 indicating a moderate positive relationship (Wang et al., 2022). Specifically, regression analysis run separately by gender indicated object control skills were a significant positive predictor of MVPA time for boys and locomotor skills were a significant positive predictor of MVPA time for girls (Wang et al., 2022). The mean age of the sample in this study was 13.3 years in comparison to 9.5 years in the current study, suggesting that the relationship between PA and motor competence may be more established in adolescence. However, further methodological differences between this study and the present study make it difficult to draw comparisons. Wang et al. (2022) measured PA using different accelerometer placement, metrics, and processing methods. Furthermore, each study employed different motor skill assessments (e.g., TGMD-2 vs BOT-2). Further research with comparable methods is therefore required to better understand the association between motor competence and PA in children with intellectual disabilities and/or autism.

Associations between motor competence and PA are potentially influenced by a range of individual, social and environmental factors (Barnett et al., 2016; Foweather et al., 2015; Niemistö et al., 2019). It may be that other socio-ecological factors are more likely to explain a variance in PA levels within this population more so than motor competence does. A review by Sutherland and colleagues (2021) highlighted that research involving children with intellectual disabilities has predominantly investigated factors associated with PA behaviours at an intrapersonal level, but the majority show no significant association with PA (Sutherland et al., 2021). Within the socioecological model, outside of the intrapersonal level, facilities, resources and adapted PA programmes for children and adolescents with intellectual disabilities available in the community have been identified as key factors influencing the PA participation at the environmental level (Yu et al., 2022). This highlights the need for accessible facilities and resources to provide a basic guarantee for PA participation of children and adolescents with intellectual disabilities (Yu et al., 2022). Considering the relatively high activity levels of participants in the current study it is plausible that being active is possible for children with intellectual disabilities and/or ASD regardless of motor competence level. Quality of movement may be less important within inclusive leisure-time PA opportunities due to a focus on participation rather than competition and sport. This is comparable to the relationship between weight and PA in children and adolescents with intellectual disabilities, as data would appear to suggest that weight status is less relevant to PA participation in this population with overweight/obese children with intellectual disabilities not less likely to participate in PA in comparison to their overweight/obese TD peers (Sutherland et al., 2021).

Overall motor competence levels in this study most commonly fell within the below average and average ranges in comparison to TD peer data norms. Previous research using the BOT-2 has differed in finding impairments on all subtests within this population. For example, in a sample of 53 children with ASD between the ages of 7-14 years and a sample of 119 children with ASD aged 6-12 years results were well below average or below average in comparison to TD peer data norms (Alsaedi, 2020; Liu et al., 2021). Of the six subtests assessed in this study, upper limb coordination and strength were consistently the weakest. Upper limb coordination of the BOT-2 assessment includes skills focused on throwing and catching. In a study of students with varying degrees of intellectual disabilities, Jeoung (2013) found the skill of throwing to be the weakest of all object manipulation skills assessed. Explanations for particularly poor performance of throwing and other general object control skills in this group of children include the nature of their complexity and being an open skill which relies on environmental factors (Kavanagh et al., 2023). Additionally, there may be greater involvement of cognitive functions and processes required to successfully execute these skills which children with intellectual disabilities experience deficits in (Kavanagh et al., 2023). Wuang et al. (2013) assessed lower-limb muscle strength in children with and without intellectual disabilities and identified the children with intellectual disabilities as having significantly reduced muscle strength in lower-limbs compared to the TD children of the sample. In TD peers strength has been shown to be important for health outcomes (García-Hermoso et al., 2019). Given the health inequalities experienced by children and young people with intellectual disabilities (Emerson and Spencer, 2015), and the poor performance in strength tests demonstrated in this study, suitable strength-based activities should be encouraged to improve health.

Based on new UK PA guidelines for disabled children and young people (Department of Health and Social Care UK, 2022), children in the current study were sufficiently active overall, with only two participants not achieving the recommended 120-180 minutes of MVPA per week. These are the first guidelines designed specifically for disabled children and young people and are based on a rapid evidence review which concluded that there was little evidence to support the recommendations that disabled children and young people should engage in a weekly average of 60 minutes per day of MVPA, as per the WHO and USA guidelines (Smith et al., 2022). When considering the 60 minutes per day guidelines, less than half (42%) of the children in the current study met these, although the average daily value for the whole group was less than 4 minutes away from this threshold (56.7 minutes). Moreover, in the current study on average participants engaged in significantly more MVPA on weekdays (59.5min ± 22.2) compared to weekend days (50.1min ± 22.6). These findings are in line with other studies involving youth with disability which have demonstrated that PA is lower during weekend days compared to weekdays and that the school setting is where most activity is accrued, thus supporting the significant of environmental factors for PA participation (Eguia et al., 2015; Liang et al., 2020).

Average acceleration of the participants in the current study (60.7 mg) was higher in comparison to previously published data in TD peers of a similar age (45.4 mg; 9-10 years; Fairclough et al., 2019) and in TD adolescent girls (36.3 mg; Rowlands et al., 2018). Conversely, the IG was steeper (-2.07) in comparison to TD 9–10-year-olds (-1.96) in Fairclough et al. (2019) but shallower than the TD adolescent girls (-2.47) reported in Rowlands et al (2018). Higher average acceleration in comparison to TD peers indicates a greater volume of PA whereas a steeper IG in comparison to TD peers of a similar age (9-10 years) indicates lower intensity PA. Higher intensity PA is of greatest benefit for health (Aadland et al., 2018; Fairclough et al., 2017), and it has been demonstrated that PA intensity is more important than PA volume for cardiorespiratory fitness (Fairclough et al., 2019). Additionally, an increase in IG (becoming shallower, less negative) is associated with a favourable decrease in BMIz and waist to height ratio (Fairclough et al., 2019). It has been previously reported that children with intellectual disabilities engage in less continuous bouts of PA and that bouts decrease as the intensity of activity increases (Downs et al., 2016). The intensity at which children with intellectual disabilities and/or ASD engage in activity appearing to be lower than their TD peers highlights another area of targeted need.

Strengths of this study include the methods used. The BOT-2 motor competence tool is psychometrically appropriate for use amongst children with intellectual disabilities and ASD (Downs et al., 2020). The analyses of raw wrist worn accelerometer data is also a strength of the study, particularly the novel cut-point free analyses providing an opportunity in the future for comparison across studies using raw acceleration outcomes. This study was not without limitations. Firstly, categorisation of motor competence was based on TD normative data which may lead to error as children with intellectual disabilities develop motor related skills at a slower rate than TD children (Alesi et al., 2018). Using standardised norms to interpret performance may subsequently increase the likelihood of below average scores due to this slower development rate. Specific classifications for this population in the future research which account for different developmental trajectories could improve accuracy. In relation to the collection of PA data, accelerometers did not monitor water-based activity and therefore we must note that PA levels may have been underestimated as some popular activities (e.g., swimming; Allen et al., 2022) were not captured. Additionally, the cut-points used to classify some of the PA outcomes are not population specific. Future research is needed to understand if the values are appropriate within this population. Though a move to cut-point free analyses in future research is recommended, classification of MVPA levels using cut-points allows researchers to understand if children with intellectual disabilities and/or ASD are achieving the recommended PA guidelines. Work to establish population specific cut-points would allow for a more accurate comparison against these guidelines. Measurement of activity was also across two time periods of November to January and March to July, and adjustment for seasonal variation was not possible due to insufficient power to adjust for further covariates. In relation to the study sample, the sex ratio was uneven and the sample size was smaller than intended reducing the statistical power for the analyses. Issues relating to uneven sex ratios (McKenzie et al., 2016) and small sample sizes (Hinckson and Curtis, 2013) are commonly reported in research involving this population, particularly when measuring PA. The small sample also meant that associations between PA and motor competence are reported for a heterogeneous group in which children with intellectual disabilities, intellectual disabilities and ASD, and ASD only were combined for analyses. Though adding disability type and disability severity as covariates to the regression models did not significantly improve the model fit and therefore findings appear to be consistent, irrespective of disability.

## Conclusion

In conclusion, no associations were found in this study between PA outcomes and motor competence. Overall, PA levels were lowest on weekend days, and engagement in high PA intensity across the whole week, as demonstrated with the IG outcome, was poor in comparison to data from TD peers. Many children in the current study reported below average scores for individual motor competence subtests, although average scores were also evident. To help better understand the relationship between PA and motor competence amongst children with intellectual disabilities and ASD more longitudinal studies are required including large sample sizes to allow for subgroup analysis of different disability types and severity. Furthermore, it is important that research with disabled children and young people considers the influence and impact of wider environmental factors that may be of greater importance for PA participation in this group in comparison to intrapersonal factors. Interventions to improve motor skill performance are warranted.
